# The Role of Attachment Styles on Quality of Life and Distress Among Early-Stage Female Breast Cancer Patients: A Systematic Review

**DOI:** 10.1007/s10880-023-09940-w

**Published:** 2023-02-11

**Authors:** Spyridoula Karveli, Petros Galanis, Eirini Marina Mitropoulou, Evangelos Karademas, Christos Markopoulos

**Affiliations:** 1 School of Medicine, National and Kapodistrian University of Athens, 75 Mikras Asias Str, 11527 Athens, Greece; 2 Clinical Epidemiology Laboratory, Faculty of Nursing, National and Kapodistrian University of Athens, Athens, Greece; 3 Department of Psychology, Faculty of Social Sciences, University of Crete, Rethymnon, Greece

**Keywords:** Attachment dimensions, Breast cancer survivors, Psychological distress, Quality of life

## Abstract

Cancer patients’ quality of life (QoL) and distress are affected by dispositional factors such as attachment anxiety or avoidance. In this review, we aimed to provide a thorough overview of the relationship between attachment dimensions and QoL and distress among early-stage breast cancer patients. Following PRISMA guidelines, we conducted a systematic search using PubMed, PsycINFO, Scopus, Cinahl, Google Scholar, and PMC Europe. We reviewed 8 eligible studies describing 1180 patients. Insecure attachment appeared to be related to poorer QoL and higher distress levels. Avoidant attachment was more frequent and was more often associated with more negative outcomes. Healthcare providers should consider investigating modifiable personality traits in the immediate post diagnosis period to identify patients more vulnerable to mental health problems, deliver personalized care, and reduce emotional burden.

**Supplementary Information:**

The online version contains supplementary material available at 10.1007/s10880-023-09940-w.

Situations like being faced with a breast cancer diagnosis and treatment require considerable adjustment and the ability to cope. Women are called to cope with survival concerns and the fear of the disease’s recurrence and death (Luz et al., 2020), premature menopause leading to the loss of fertility (Anders et al., 2009), disruptions in professional and daily routines due to the barriers posed by treatments (Keesing et al., 2018), altered body image (Falk Dahl et al., 2010), and impaired sexual functioning (Dow & Kennedy Sheldon, 2015). The most common consequences of breast cancer include depression (Burgess et al., 2005; Pilevarzadeh et al., 2019), anxiety (Hashemi et al., 2019), post-traumatic stress disorder (Hegel et al., 2006), impaired health-related quality of life (Montazeri, 2008) and moderate to severe distress (Hegel et al., 2006; Mertz et al., 2012). In comparison to the general female population, the prevalence of depression, anxiety, or both among early-stage breast cancer patients is double, especially during the first year after diagnosis (Burgess et al., 2005).

From diagnosis to survivorship, health-related QoL and psychological distress are increasingly considered critical outcomes. The American Society of Clinical Oncology guidelines recognize the importance of distress screening and propose that this should be included in oncology settings to holistically treat a patient (Smith et al., 2018b). Likewise, the American College of Surgeons’ Commission on Cancer (ACSCoC, 2020) includes psychosocial distress screening in the accreditation process. Distress, which “…extends along a continuum, ranging from common normal feelings of vulnerability, sadness, and fears to problems that can become disabling, such as depression, anxiety, panic, social isolation, and existential and spiritual crisis” (Riba et al., 2019, p. 2) is considered to have a negative impact on how patients cope with the disease, its symptoms, and its treatment. It has also been found that distress has a negative impact on all QoL domains (Costa-Requena et al., 2012); it is often accompanied by psychiatric disorders such as adjustment disorder, depression, and anxiety disorders (Andrykowski et al., 2002; Coyne et al., 2004), while unmanaged distress affects cancer morbidity and mortality (Institute of Medicine [IOM]; Adler & Page 2008). Accordingly, health-related QoL is regarded an essential outcome measure in oncology clinical trials (Montazeri, 2008).

Some breast cancer survivors are more distressed or have poorer QoL than others. Distress is predicted by contextual factors such as breast cancer stage, treatment, and cancer recurrence; treatment-related factors, such as menopause, type of surgery, and fatigue; sociodemographic characteristics, like younger age and being single; comorbidities, such as past history of mental health illness and functional limitations; and emotional regulation factors, such as lower social support and smoking (Syrowatka et al., 2017). Similar factors have been found to predict a poor QoL in cancer patients (Mokhatri-Hesari & Montazeri, 2020; Montazeri, 2008).

Most of the above-mentioned factors are either not modifiable (e.g., stage, type of treatment, age) or affected by personality characteristics. Consequently, dispositional factors have received greater attention in recent years as key determinants of adjustment to chronic disease (Stanton & Revenson, 2011). Attachment theory suggests that certain attachment styles are associated with greater vulnerability to distress in the face of adverse life events (Mikulincer & Shaver, 2017). John Bowlby (1969, 1973, 1980) supported the idea that humans instinctively form close emotional bonds to survive. The independent biological system of attachment is activated during times of threat, pain, fatigue, and sickness to increase an infant’s chances of survival by motivating proximity-seeking behaviors such as smiling, crying, and vocalizing to alert a stronger, protective figure (Bowlby, 1973). The attachment figure’s availability and responsiveness to these behaviors will subsequently have an important impact not only on survival but on emotional regulation as well. Depending on the attachment figure’s degree of availability, the quality of their responsiveness and the success or failure of the primary attachment strategy to achieve the security-set goal, the child develops mental representations about the self and others, which are known as internal working models. These models are stored and “…provide the cognitive skeleton of a person’s attachment orientation” (Mikulincer & Shaver, 2017, p. 147) in later relationships.

In adults, two dimensions capture the ways that a person approaches close others while under duress: attachment anxiety and attachment avoidance (Fraley et al., 2015; Mikulincer & Shaver, 2007). These dimensions are used to characterize attachment style, that is, a consistent set of ways of regulating emotion, particularly in terms of attention to and expression of emotion, appraisals of threat, and the use of social coping resources (Fuendeling, 1998). Anxious attachment entails a pattern of hyperactivation that is characterized by heightened distress, exaggeration of the negative, the persistent seeking of help and reassurance from others, and worry about a partner’s availability and one’s value to their partner (Mikulincer & Shaver, 2007). On the other hand, avoidant attachment entails a deactivation pattern that is characterized by the minimization of distress, discomfort with closeness and dependence on relationship partners, a tendency to be overly self-reliant, the avoidance of proximity-seeking, and avoidance of whatever triggers threats (Mikulincer & Shaver, 2007).

Attachment theory, which has been applied to the study of emotion regulation, provides a valuable framework for comprehending individual differences in affect regulation processes (Mikulincer et al., 2003). Individual differences in attachment have an impact on the way patients appraise a disease, the way they attend to and express their emotions, as well as in the coping strategies they employ. Awareness of a patient’s specific attachment orientation can help health care providers understand the pathway to high distress and consequently, to apply interventions tailored to the individual’s needs, so as to restore security and relieve distress.

A recent meta-analysis revealed a positive association between insecure attachment (avoidant and anxious attachment), depressive symptoms, and anxiety, and a negative association with social support (Nissen, 2016), in cancer patients. These results are consistent with the findings of a prior systematic review which was conducted by Nicholls et al. (2014); these researchers also found that the anxiety dimension was associated with depression, higher anxiety, lower levels of social well-being and poorer well-being. Avoidant attachment was also associated with higher levels of depression, poorer well-being and poor perceived QoL. Other studies examining the impact of attachment on psychosocial outcomes in mixed-stage breast cancer patients have found a positive association between attachment anxiety and distress (Arambasic et al., 2019; Favez et al., 2016; Ouakinin et al., 2015) or depression (Hsiao et al., 2013).

The above-mentioned systematic reviews (Nicholls et al., 2014; Nissen, 2016) on cancer and attachment have certain limitations, including lack of focus on a particular cancer type and the mixed stage of cancer samples. Also, Nissen’s (2016) meta-analysis incorporated primarily terminally ill patients. Therefore, both reviews treat cancer patients as one group, regardless cancer type or stage, probably due to the small number of available studies. The prevalence of psychological distress varies among cancer types (Derogatis et al., 1983; Zabora et al., 2001) and different cancer stages (Reich et al., 2007). Therefore, it would be erroneous to perceive cancer patients as a homogeneous group. Moreover, past reviews have not considered how gender may influence emotional responses, adjustment, and coping following an illness (Bogg et al., 2000) and attachment dimensions (Scharfe, 2016). A recent meta-analysis on sex differences in the avoidance and anxiety dimensions of adult romantic attachment revealed that males in community groups were higher in avoidance and lower in anxiety in comparison to females (Del Giudice, 2011). Given that breast cancer is the most common cancer among women, and at the time of diagnosis 94% of patients are diagnosed with non-metastatic disease (American Cancer Society [ACS], 2019), it is important to have a clear picture regarding the profile of the majority of patients diagnosed with breast cancer.

For the reasons described above, we focused the present review on early-stage female breast cancer patients and aimed to provide a thorough overview of the relationship between attachment dimensions (anxiety and avoidance) and QoL and distress. In a medical setting, the identification of the patient’s attachment orientation early on in the diagnosis process could provide significant information to interdisciplinary team members (e.g., breast surgeons, oncologists, oncology nurses) who may not be so familiar with the importance of this concept. First and foremost, individuals who are at risk of poor adjustment can be identified by mental health professionals and receive adequate care at an early point, lessening the emotional strain of the disease. On the other hand, health care providers will be able to better understand their patients' reactions to stress, communication and interaction needs, and the level of compassion and support required, and so tailor their approach to fit their patients' individualized needs.

## Method

A systematic review protocol was published on the International Prospective Register of Systemic Reviews (PROSPERO, reference no. CRD42021224800). The review development and methods were in accordance with The Preferred Reporting Items for Systematic Reviews and Meta-Analyses (PRISMA; Liberati et al., 2009; Moher et al., 2009) and the Institute of Medicine’s (2011) guidelines.

### Search Strategy

PubMed, PsycINFO, Scopus, Cinahl, Google Scholar, and PMC Europe were searched for articles that were published between when the platforms were created and February 2021. The PICOS (Liberati et al., 2009) framework was used to build an appropriate search strategy. A concept map that combined medical subject headings (MeSH), terms, and keywords was also used. The main components of the search strategy included “breast cancer,” “attachment,” “quality of life,” “distress,” “depression,” and “anxiety.” The complete search strategy is provided in supplementary material 1.

To minimize selection bias, we adopted guidelines from Grey Matters (CADTH, 2018. Updated April 2019) and other sources (Godin et al., 2015) and the search was extended to dissertations or any unpublished studies that fit into the eligibility criteria. More specifically, targeted searches were conducted using OpenGrey, the *Grey Literature* report (The New York Academy of Medicine), the OSF pre-prints archive, Open Access Theses and Dissertations, EThOS (e-theses online library, British Library), CORE, and Research Gate, as well as two web search engines (www.google.gr and www.duckduckgo.com). The records that were retrieved from the latter search were screened beforehand based on their titles and abstracts, and only those that fit the eligibility criteria were uploaded to the citation management software. Moreover, in order to ensure a thorough review of the literature, supplementary search methods were used. The first author contacted experts in the field to obtain their recommendations of specific documents that were relevant to the research question or to discuss any further unpublished work that could have been included in the review. Additionally, we searched the publications of authors of relevant research using SCOPUS. Reference lists and forward citations of all the included studies were also scanned.

### Eligibility Criteria

Studies were identified based on the following inclusion criteria: 1. studies with quantitative data that was obtained through self-rated questionnaires, 2. early stage (0-III) breast cancer survivors, 3. studies with correlations between validated attachment style scales and validated measures related to patients’ QoL and/or psychological distress, 4. study populations equal or above 50 breast cancer patients, and 5. empirical articles written in English.

The decision to include solely studies using validated self-reported measures of attachment was taken to avoid inconsistencies between attachment measurements (Ravitz et al., 2010; Roisman et al., 2007). In addition, according to its administration manual, the Adult Attachment Interview should not be conducted within the context of crisis or trauma (Hesse, 2016).

Eligible records included journal articles, books and book chapters, theses and dissertations, brief reports, conference abstracts, and presentations. Furthermore, when the study sample included multiple cancer groups or healthy controls, only information about breast cancer patients and survivors was included. Studies were excluded if they met any of the following criteria: 1. a qualitative design, 2. metastatic (stage IV) breast cancer patients, and/or 3. attachment that referred to medical (e.g., cell attachment) and not to human attachment. We also excluded studies that were written in the form of systematic reviews and meta-analyses.

### Study Selection

Covidence software was used for the title, abstract screening, and full-text screening. Initially, study titles and abstracts were screened for eligibility independently by two reviewers (SK & E-MM). At this stage, the agreement between the reviewers was moderate (64%) since most disagreements were due to one reviewer having a more liberal approach regarding attachment. A third independent reviewer (PG) resolved all the discrepancies, which were also discussed with the two independent reviewers (SK & E-MM) during a consensus meeting. During the next stage, full-text articles of the remaining papers were independently assessed for inclusion by both reviewers (SK & E-MM). When there was insufficient information to determine a study’s eligibility or no statistical analyses, one reviewer (SK) contacted the author for more information. At this stage, the reviewers agreed with one another.

### Data Extraction

Covidence software was used for data extraction. Two reviewers (SK and E-MM) independently extracted the data for each full-text paper by following the Covidence structured format. The data that were extracted included the author’s name, the year of publication, the state or city and country of origin, the dates of the data collection, the study design, the sample size, the sampling method, and the measures of attachment that were used.

### Quality Assessment

We used the Joanna Briggs Institute critical appraisal tools (Santos et al., 2018) to assess the quality of the studies. These tools include a 9-point scale that is used for prevalence studies, an 8-point scale that is used for cross-sectional studies, and an 11-point scale that is used for cohort studies. In prevalence studies, a score of 8–9 indicates good quality, whereas a score of 5–7 indicates moderate quality, and a score of 4 indicates poor quality. In cross-sectional studies, a score of 7–8 indicates good quality, a score of 4–6 indicates moderate quality, and a score of 3 indicates poor quality. In cohort studies, a score of 9–11 indicates good quality, a score of 5–8 indicates moderate quality, and a score of 4 indicates poor quality.

## Results

### Identification and Selection of Studies

**Fig. 1:**
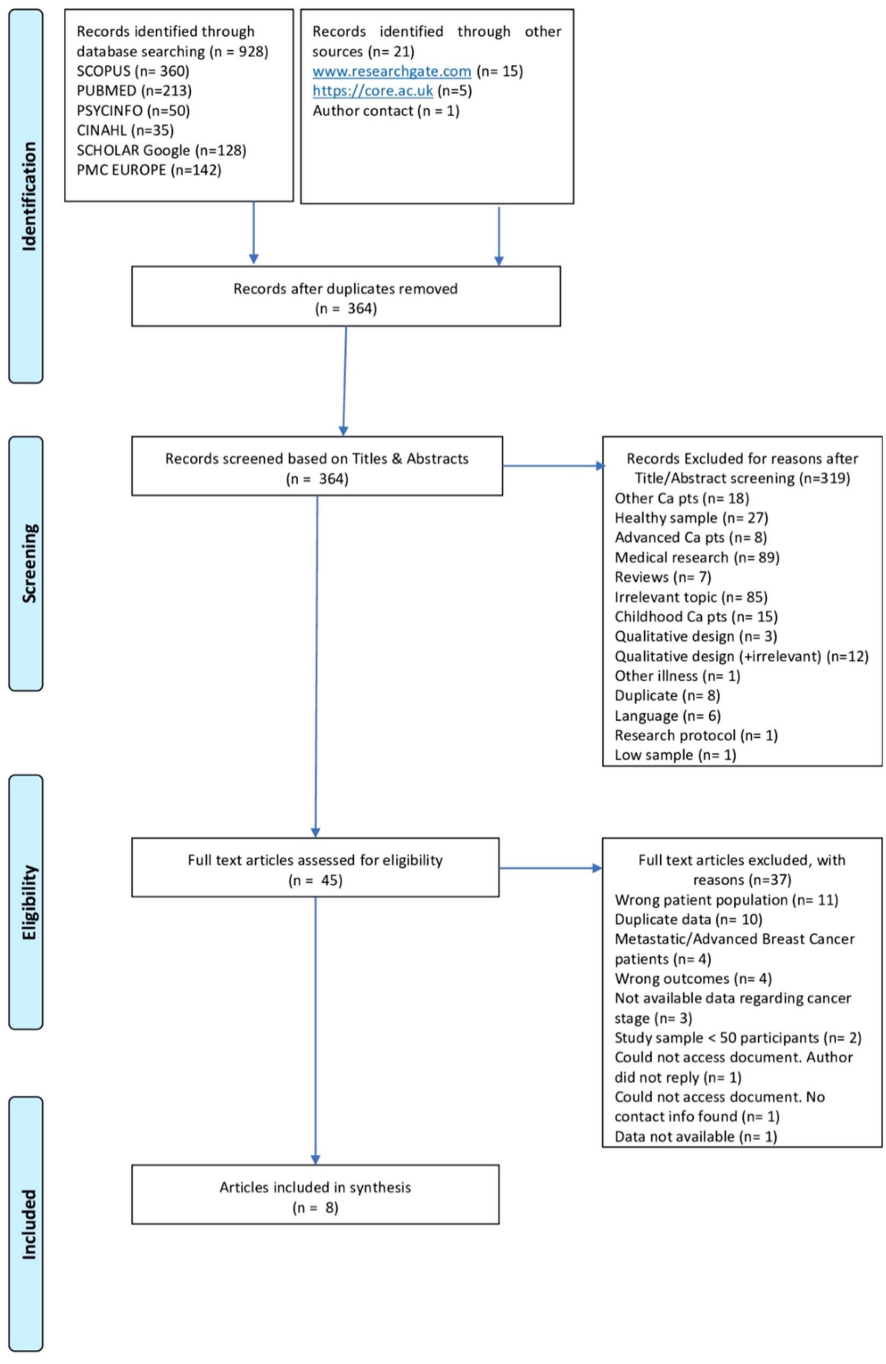
Selection of studies for inclusion in review

The flowchart that details the literature search is summarized in Fig. 1. The whole search resulted in 949 records. After removing 585 duplicates, 364 records were screened for eligibility based on their titles and abstracts. Of these records, 319 records did not meet the inclusion criteria, and, as a result, the remaining 45 full-text studies were assessed for eligibility. A total of 37 studies were excluded because (a.) the analyses contained survivors of both sexes and/or other cancer types that were not appropriately reported (*n* = 11), (b.) the study sample included metastatic or advanced breast cancer patients (*n* = 4), (c.) the sample size was below 50 participants (*n* = 2), (d.) the outcome measures were not relevant to patients’ QoL and/or psychological distress (*n* = 4), (e.) the study results were published in other publications that were included in the screening (*n* = 3), (f.) the records were duplicates (*n* = 7), and finally (g.) the cancer stage was unclear (*n* = 3). Accessing the full-text documents was not possible for 3 records. Consequently, 8 eligible studies remained.

### Characteristics of the Included Studies

The main characteristics of the studies that have been included in this systematic review are illustrated in Table 1. A total of 1,180 females who had breast cancer from 7 countries (the US, Australia, Portugal, France, Switzerland, Israel, and South Korea) were included in our review. The number of breast cancer patients in studies ranged from 58 to 348, while the mean age ranged from 47.3 to 57.5 years. The time since diagnosis ranged from 1-week post-surgery to 5 years, whereas two studies did not report information regarding the time since diagnosis (Ávila et al., 2015; Jang et al., 2015). The majority of the studies were cross-sectional (*n* = 5) and used a convenience sample, while three studies had a longitudinal design with three (Brédart et al., 2015b; Favez & Cairo Notari, 2021) and two (Lee et al., 2018) measurement points, respectively. All the studies were published in journals. Attachment, QoL, and distress were assessed using self-reported measures. In particular, the Experience of Close Relationships (ECR) questionnaire and its versions were used in all studies to assess attachment orientation. All studies except for one (Jang et al., 2015) used both subscales of the ECR (anxiety and avoidance). However, three different tools were used to estimate patients’ QoL and two to estimate distress: the World Health Organization’s Quality of Life Assessment (the short version), the Functional Assessment of Cancer Therapy-Breast scale, and the European Organization for Research and Treatment of Cancer Quality of Life Questionnaire (Breast Cancer Module) were used to assess patients’ QoL, whereas the Hospital Anxiety and Depression Scale and the Mental Health Inventory were used to assess distress. Tools measuring other QoL parameters were also utilized (Body Image Scale and Pain Intensity).

**Table 1:** Characteristics of the studies included in the review

Author	Country	Setting	Design	Dates of data collection	Sampling method	Response rate	Sample size	Age (years), Mean (SD)	Measure of Attachment	Outcome measures		Publication
										Measure of QoL	Measure of Distress	
Ávila et al. (2015)	Portugal	2 public hospitals and a nonprofit organization	CS	2011–2014	Convenience sampling	NR	127	48.94 (8.20)	Experiences in Close Relationship Scale—Short Form (ECR-12)	World Health Organization Quality of Life—Brief Assessment		Journal
Brédart et al. (2015b)	France	Hospital	L	March 2012–February 2013	Convenience sampling	T1: 85%, T2: 74%, T3: 66%	348	55.2 (12.4)	Experiences in Close Relationships Scale—Modified Short Form (ECR-M16)		Hospital Anxiety and Depression Scale (HADS)	Journal
Fagundes et al. (2014)	Ohio/the US	breast cancer clinics and media announcements	CS	NR	Convenience sampling from a different RCT	NR	96	51.83 (9.47)	Experiences in Close Relationships Scale—Modified Short Form (ECR-M16)	Functional Assessment of Cancer Therapy‐Breast (FACT‐B)		Journal
Favez and Cairo Notari (2021)	Switzerland	University Ηospital	L	NR	Convenience sampling	67.3%	110	53.3 (12.6)	Revised Experiences in Close Relationships questionnaire (ECR-R; French version)	European Organization for Research and Treatment of Cancer Quality of Life Questionnaire, Breast Cancer Module (EORTC QLQ- BR23)		Journal
Inbar et al. (2012)	Israel	Hospital	CS	NR	Convenience sampling	83%	58	48.73 (5.36)	Experiences in Close Relationships (ECR-36)-Hebrew version		Μental Health Inventory	Journal
Jang et al. (2015)	Korea	1 university hospital and 1 university medical center	CS	July 2010–June 2013	Convenience sampling	NR	199	48.1 (9.4)	Modified Experiences in Close Relationships (ECR-M36)	World Health Organization Quality of Life—Brief Assessment	Hospital Anxiety and Depression Scale (HADS)	Journal
Lee et al. (2018)	Korea	1 university hospital and 1 university medical center	L	January 2012–June 2014	Convenience sampling	58.2%	114	47.3 (9.5)	Modified Experiences in Close Relationship (ECR-M36)		Hospital Anxiety and Depression Scale (HADS)	Journal
Smith et al. (2018a)	Australia	Νon-profit organization	CS	NR	Voluntary response sampling	18.3%	128	57.5 (8.9)	Modified Experiences in Close Relationships (ECR-M36)	Functional Assessment of Cancer Therapy‐Breast (FACT‐B)		Journal

### Quality Assessment

The quality assessment of the studies that have been included in our review is presented in Table 2. The quality was good for five studies (Ávila et al., 2015; Fagundes et al., 2014; Favez & Cairo Notari, 2021; Lee et al., 2018; Smith et al., 2018a) and moderate for three studies (Brédart et al., 2015b; Inbar et al., 2012; Jang et al., 2015). Poor identification of the confounding factors and the limited application of multivariable methods to eliminate these factors were the main limitations of the studies.

**Table 2:** Quality of studies included in this systematic review

	Ávila et al. (2015)	Brédart et al. (2015b)	Fagundes et al. (2014)	Favez and Cairo Notari (2021)	Inbar et al. (2012)	Jang et al. (2015)	Lee et al. (2018)	Smith et al. (2018a)
1. Were the criteria for inclusion in the sample clearly defined?	X	X	X	X		X	X	X
2. Were the study subjects and the setting described in detail?	X	X	X	X	X	X	X	X
3. Was the exposure measured in a valid and reliable way?	X	X	X	X	X	X	X	X
4. Were objective, standard criteria used to measure the condition?	X	X	X	X	X	X	X	X
5. Were confounding factors identified?	X		X	X			X	X
6. Were strategies for dealing with confounding factors stated?	X		X	X			X	X
7. Were the outcomes measured in a valid and reliable way?	X	X	X	X	X	X	X	X
8. Was appropriate statistical analysis used?	X	X	X	X	X	X	X	X
Total quality	Good	Moderate	Good	Good	Moderate	Moderate	Good	Good

### Measures of Attachment

The studies that have been included in this review were restricted to those that used self-report attachment measures. All of the studies that were included in the synthesis relied on the Experiences in Close Relationships scale (ECR [Brennan et al., 1998]) and its modified versions (ECR-R [Fraley et al., 2000], ECR-12 [Lafontaine et al., 2015], ECR-M16/M36 [Lo et al., 2009]) to measure the degree to which dimensions of attachment anxiety (a negative sense of self) and attachment avoidance (a negative sense of others) were present.

### Narrative Synthesis of Findings

#### Impact of Attachment on QoL and Distress

All the studies found a negative relationship between attachment dimensions (anxiety and avoidance) and QoL and a positive relationship between these dimensions and distress. In particular, breast cancer patients with higher attachment anxiety reported a poorer overall QoL (Fagundes et al., 2014; Smith et al., 2018a), a decrease in other QoL indicators such as body image and self-esteem (Ávila et al., 2015; Favez & Cairo Notari, 2021), low satisfaction with personal relationships (Ávila et al., 2015), and heightened and persistent pain (Smith et al., 2018a). Moreover, attachment anxiety was significantly associated with higher distress levels and lower levels of well-being (Inbar et al., 2012).

Similar to results for attachment anxiety, increased attachment avoidance was associated with poorer overall QoL (Fagundes et al., 2014) and a decrease in QoL indicators such as body image (Ávila et al., 2015; Favez & Cairo Notari, 2021), sexual activity (Favez & Cairo Notari, 2021), and satisfaction with personal relationships (Ávila et al., 2015). Additionally, higher levels of attachment avoidance were associated with worse sense of well-being (Inbar et al., 2012), higher levels of anxiety and depression (Lee et al., 2018), and more side effects due to treatment (Favez & Cairo Notari, 2021). In accordance with these results, Jang et al. (2015) used the overall ECR score and found that patients having more secure attachment predicted lower levels of anxiety and depression and a better QoL.

Longitudinal design studies provide a better insight on the relationship of the concepts under investigation. More specifically, Lee et al. (2018) aimed to identify the factors present at the initial breast cancer treatment stage that were associated with unresolved distress at the 1-year follow-up after breast cancer surgery. According to their findings, 46.5% of the patients were significantly distressed at the baseline level, and of these, 58.5% remained in the distress group at the 1-year follow-up. Attachment avoidance and level of distress at baseline were significantly associated with psychological distress at 1-year follow-up. Moreover, Lee et al.’s findings support the stability of attachment dimensions over time since they found no significant change in attachment insecurity (including anxiety and avoidance subscales) between the baseline and the 1-year follow-up.

Brédart et al. (2015b), who aimed to identify patterns of simultaneous trajectories (changes in questionnaire scores over the 8 months following treatment completion), reported that 32% of the patient sample who belonged to the “borderline group” (presenting possible sub-clinical anxiety and depression), and 5% of the patient sample who belonged to the “chronic distress group” (since they presented possibly clinical anxiety trajectories and mostly higher depression trajectories than the other sub-groups), were characterized by higher insecure attachment. More specifically, those in the borderline group exhibited higher scores for insecure attachment, especially the anxious dimension, whereas the chronic distress group was characterized by high scores in both the anxious and avoidant attachment dimensions.

Finally, Favez and Cairo Notari (2021), who investigated the association of attachment dimensions with QoL indicators such as body image and self-esteem, sexual activity, and treatment side effects 2 weeks, 2 months and 12 months after surgery, found that attachment anxiety and avoidance were negatively associated with body image perceptions. Additionally, at each time point, attachment avoidance predicted not being sexually active.

Values in Table 3 suggest that attachment avoidance was marginally more strongly endorsed than attachment anxiety among studies of early stage breast cancer survivors. In five (Brédart et al., 2015b; Fagundes et al., 2014; Jang et al., 2015; Lee et al., 2018; Smith et al., 2018a) out of seven studies, women had higher scores for attachment avoidance, whereas in the other two studies (Ávila et al., 2015; Favez & Cairo Notari, 2021), they reported higher on attachment anxiety. It should be mentioned that the number of patients scoring higher either on avoidant or anxious attachment dimension was not provided by any author. As a result, it was not possible to make statistical comparisons between these two dimensions and to investigate whether there were any statistically significant differences between them.

**Table 3:** Attachment dimensions (means and SDs) among early stage breast cancer survivors

Reference	Measure (range)	Attachment anxiety Mean (SD)	Attachment avoidance Mean (SD)
Brédart et al. (2015b)	ECR-16 (1–7)	3.0 (1.3)	3.2 (1.1)
Ávila et al. (2015)	ECR-12 (1–7)	2.84 (1.56)	2.35 (1.49)
Smith et al. (2018a)	ECR-M16 (1–7)	2.9 (1.5)	3.0 (1.2)
Favez and Cairo Notari (2021)	ECR-R (1–7)	3.00 (1.13)	2.56 (1.07)
Inbar et al. (2012)	ECR (1–7)	NR	NR
Jang et al. (2015)	ECR-M36 (18–126)	54.8 (14.1)	58.1 (14.1)
Lee et al. (2018)	ECR-M36 (18–126)	53.3 (13.9)	55.9 (12.4)
Fagundes et al. (2014)	ECR-M16 (8–56)	20.37 (8.88)	21.54 (8.36)

The response scale range is the same across all studies (1–7). Fagundes et al. (2014), Jang et al. (2015) and Lee et al. (2018) have multiplied the number of items per each attachment subscale with the minimum and maximum score

*NR* not reported, *SD* standard deviation

Detailed results from the eight studies regarding the association of attachment with QoL and distress are presented in Table 4. Overall, four studies measured correlation coefficients with values ranging from 0.12 to 0.62, which demonstrated a low-to-moderate correlation (Ávila et al., 2015; Fagundes et al., 2014; Jang et al., 2015; Smith et al., 2018a). Two studies measured coefficient beta regressions (Favez & Cairo Notari, 2021; Inbar et al., 2012); one study measured odds ratios (Lee et al., 2018), and one study (Brédart et al., 2015b) compared mean values.

**Table 4:** Impact of attachment on quality of life and distress

Reference	Relation between													
Smith et al. (2018a)	ECR-16 avoidance scale and FACT-B overall scale	ECR-16 avoidance scale and FACT-B physical scale	ECR-16 avoidance scale and FACT-B social scale	ECR-16 avoidance scale and FACT-B emotional scale	ECR-16 avoidance scale and FACT-B functional scale	ECR-16 avoidance scale and FACT-B breast cancer scale	ECR-16 anxiety scale and FACT-B overall scale	ECR-16 anxiety scale and FACT-B physical scale	ECR-16 anxiety scale and FACT-B social scale	ECR-16 anxiety scale and FACT-B emotional scale	ECR-16 anxiety scale and FACT-B functional scale	ECR-16 anxiety scale and FACT-B breast cancer scale	ECR-16 avoidance scale and average pain intensity	ECR-16 anxiety scale and average pain intensity
	*r* = − .47 (*p* < .001), *b* = 0.55 (95% CI 0.35 to 0.87, * p* < .01)	*r* = − .24 (*p* < .01), * b* = 1.07 (95% CI 0.72 to 1.59)	*r* = − .59 (*p* < .001), * b* = 0.39 (95% CI 0.25 to 0.6, * p* < .001)	*r* = − .26 (*p* < .01), * b* = 0.96 (95% CI 0.68 to 1.37)	*r* = − .4 (*p* < .001), * b* = 0.7 (95% CI 0.47 to 1.05)	*r* = − .12, * b* = 0.94 (95% CI 0.64 to 1.37)	*r* = − .62 (*p* < .001), * b* = 0.47 (95% CI 0.31 to 0.72, * p* < .001)	*r* = − .28 (*p* < .01), * b* = 0.85 (95% CI 0.69 to 1.36)	*r* = − .66 (*p* < .001), * b* = 0.43 (95% CI 0.3 to 0.61, * p* < .001)	*r* = − .43 (*p* < .01), * b* = .7 (95% CI 0.52 to 0.95, * p* < .05)	*r* = − .56 (*p* < .001), * b* = 0.51 (95% CI 0.34 to 0.76, * p* < .01)	*r* = − .2 (*p* < .05), * b* = 0.96 (95% CI 0.69 to 1.33)	*r* = .26 (*p* < .01)	*r* = .22 (*p* < .05)
Ávila et al. (2015)	ECR avoidance scale and WΗO-QoL physical scale	ECR avoidance scale and WΗO-QoL psychological scale	ECR avoidance scale and WΗO-QoL social relationships scale	ECR anxiety scale and WΗO-QoL physical scale	ECR anxiety scale and WΗO-QoL psychological scale	ECR anxiety scale and WΗO-QoL social relationships scale								
	*r* = − .17	*r* = − .28 (*p* < .01)	*r* = − .32 (*p* < .001)	*r* = − .01	*r* = − .23 (*p* < .01)	*r* = − .27 (*p* < .01)								
Favez and Cairo Notari (2021)	ECR avoidance scale and EORTC QLQ-BR23 side effects score of treatment	ECR anxiety scale and EORTC QLQ-BR23 side effects score of treatment	ECR avoidance scale and body image scale	ECR anxiety scale and body image scale										
	*b* = 0.05 (95% CI − 0.02 to 0.12, * p* = .13)	*b* = 0.03 (95% CI − 0.04 to 0.09, * p* = .45)	*b* = 1.88 (95% CI 0.95 to 2.8, * p* < .001)	*b* = 1.29 (95% CI 0.43 to 2.16, * p* = .003)										
Inbar et al. (2012)	ECR avoidance scale and MHI (mental well-being scale)	ECR avoidance scale and MHI (mental distress scale)	ECR anxiety scale and MHI (mental well-being scale)	ECR anxiety scale and MHI (mental distress scale)										
	*b* = − 0.26 (*p* < .01)	*b* = 0.11	*b* = − 0.32 (*p* < .001)	*b* = 0.45 (*p* < .001)										
Jang et al. (2015)	ECR-36 overall scale and HADS anxiety scale	ECR-36 overall scale and HADS depression scale	ECR-36 overall scale and WΗO-QoL overall scale											
	*r* = .29 (*p* < .001)	*r* = .34 (*p* < .001)	*r* = − .4 (*p* < .001)											
Fagundes et al. (2014)	ECR-16 avoidance scale and FACT-B total score	ECR-16 anxiety scale and FACT-B total score												
	*r* = − .31 (*p* < .01), * b* = − 0.41 (95% CI − 0.84 to 0.02, * p* = .06)	*r* = − .39 (*p* < .01), * b* = − 0.59 (95% CI − 0.99 to − 0.18, * p* = .01)												
Lee et al. (2018)	ECR-36 avoidance scale and HADS overall scale													
	O* r* = 1.045 (95% CI 1.002 to 1.09, * p* = .038)													
Brédart et al. (2015b)	ECR-16 avoidance scale and profiles	ECR-16 anxiety scale and profiles												
	Mean for supported = 2.7, resilient = 3.3, borderline distress = 3.6, and chronic distress = 4 (*p* < .001)	Mean for supported = 2.7, resilient = 2.4, borderline distress = 3.6, and chronic distress = 4.7 (*p* < .001)												

*b* coefficient beta regression, *CI* confidence interval, *MHI* mental health inventory, *OR* odds ratio, *p* p-value, *r* correlation coefficient

## Discussion

### Summary of Findings

In this review we aimed to summarize the evidence for how attachment related to QoL and distress among early-stage female breast cancer patients. We found that attachment insecurity, which was expressed either through anxiety or avoidance dimensions, was negatively related to patients’ QoL and positively related to distress. These finding were supported by both cross-sectional (*n* = 5) and longitudinal study designs (*n* = 3). The results of the latter studies revealed that attachment insecurity may play an important role in the emergence and persistence of distress since higher levels of attachment avoidance at the baseline were significantly associated with distress a year later (Lee et al., 2018) and with trajectories reflecting poorer psychological adjustment (Brédart et al., 2015b).

### Comparison with the Current Literature

Findings of the present systematic review are in line with the findings of previous studies (Nicholls et al., 2014; Nissen, 2016) that investigated the association between attachment insecurity and psychosocial outcomes in mixed cancer types and mixed stages populations. Nevertheless, it seems likely that more negative outcomes, such as persistent distress, anxiety and depression, are more often associated with attachment avoidance. Individuals characterized as highly avoidant, tend to have higher and more persistent distress levels over time (Brédart et al., 2015b; Lee et al., 2018); other researchers have also demonstrated that attachment avoidance can relate to negative emotional reactions to various health conditions (Gick & Sirois, 2010; Mula et al., 2016). Mikulincer and Shaver (2017, p. 206) proposed that “…under chronic, demanding stressful conditions, avoidant deactivating strategies seem to collapse, causing avoidant people to have even higher levels of distress than anxious people.”

Moreover, our findings reveal that there is not a clearly dominant insecure attachment dimension among early-stage breast cancer patients. Patients seemed to be slightly more likely to endorse avoidant attachment than anxious attachment, though we did not test whether this difference was statistically significant. Other researchers (Arambasic et al., 2019; Brédart et al., 2015a; Hsiao et al., 2013) have also found that attachment avoidance is slightly more frequent among breast cancer patients than attachment anxiety. Likewise, Tacón et al.’s (2001) study assessed women with and without breast cancer found that women in the breast cancer group scored significantly higher than the non-cancer group on attachment avoidance.

### Study Strengths

This systematic review is the first to examine the relationship between insecure attachment dimensions and patients’ QoL and distress among early-stage female breast cancer survivors. This focus mitigates the potentially confounding influence of gender, tumor type, and cancer stage. Given that early breast cancer is the most commonly diagnosed cancer in women, and typically at earlier stages of the disease, it is essential to focus on this target population. Finally, the a priori registration in PROSPERO and the methodology followed are among the strengths of this review.

### Study Limitations

One should interpret these findings with several limitations in mind. A significant limitation of the current review is the small number of studies that investigated the role of attachment on patients’ QoL and distress that were identified for this review (*n* = 8). This is largely due to the exclusion of studies on mixed-stages breast cancer patients. Three (Brédart et al., 2015b; Inbar et al., 2012; Jang et al., 2015) out of eight studies were of moderate quality due to the poor identification of the confounding factors and the limited application of multivariable methods to eliminate these factors. Therefore, several confounders (e.g., socioeconomic status, educational level, etc.) could affect the relation between attachment and quality of life and distress. Further studies should apply multivariable models to eliminate possible confounders and find more valid results.

The instrument used to measure attachment and distress was the same among the majority of the studies, which reflects a consensus on the definition and measurement of these concepts. In contrast, variations were found in the measurement of patients’ QoL. Another limitation is the heterogeneity of methodologies and statistical analyses, which contributed to our inability to conduct a meta-analysis and, consequently, to a lack of statistical analyses. Finally, since most of the studies in our review were cross-sectional, we cannot infer a causal relationship between attachment and quality of life.

### Recommendations for Future Research

Our findings highlight the need to investigate the changes in overall QoL among early-stage breast cancer patients and the impact of attachment over time. Accordingly, more longitudinal studies with larger samples must be conducted to explore causalities and reach more reliable conclusions regarding insecure attachment dimensions’ persistence and long-term impact (beyond 1 year) and explore the pathway to this impact through the investigation of mediating factors.

Moreover, the studies included in our review suggest that anxiously or avoidantly attached breast cancer patients are at risk for suboptimal adjustment. It is imperative that attachment-based interventions be designed and implemented to investigate their effects on patients’ quality of life and levels of distress. More specifically, it may be helpful to screen for attachment orientations and compare the short- and long-term psychological outcomes of breast cancer patients who receive multidisciplinary (e.g., surgeon, oncologist, oncology nurses), attachment-specific treatment early on in the diagnosis phase to the outcomes of breast cancer patients in a control group who receive standard multidisciplinary treatment (treatment as usual). Such an attachment-based intervention would entail providing health care practitioners with a brief training regarding attachment theory, attachment patterns and the differing needs and behaviors of patients according to their attachment style.

### Clinical Implications

Healthcare providers should consider assessing individual patients’ attachment orientation. Knowledge of a patient’s insecure attachment pattern could function as a guide for deciding communication and working alliance strategies. In particular, in the immediate post diagnosis period, patients with high attachment anxiety, who use a hyperactivation pattern, would benefit more from care that is balanced between support and autonomy and is characterized by more direction, reassurance, and a compassionate approach. On the other hand, patients with high attachment avoidance, who use a deactivation pattern, would likely benefit more from information regarding their disease and its management, as well as psycho-education regarding distress management. For instance, patients who appear extremely self-reliant and cope with their diagnoses in an overly detached, emotion-free manner should be treated with caution, since this behavior could mask an avoidant attachment orientation. Consistent with this assumption, the findings of a study (Clover et al., 2015) revealed that 71% of oncology patients screened with significant distress declined help, with the most common barriers being a preference for self-help and a belief that distress is not severe enough to warrant intervention.

Attachment insecurity may also trigger behaviors (e.g., smoking and drinking) that increase the risk of illness, interfere with health care (e.g., avoidance or overuse), and interfere with patients’ relationships with physicians and the multiple benefits of social support (Maunder & Hunter, 2015). Consequently, the early identification of a risk factor such as a patient’s insecure attachment pattern would help healthcare practitioners reduce patients’ self-sabotaging behaviors and unneeded health visits, improve adherence to medical advice, and promote functional coping strategies.

The focus of mental health professionals should be shifted to mediating factors, such as illness appraisals and coping strategies. Their focus needs to be on identifying patients’ cognitive distortions regarding their illness and self, challenging them and promoting rational thinking, identifying and replacing maladaptive coping strategies with more functional ones, while at the same time understanding the underlying unmet needs that are satisfied by the usage of these strategies. In the long run, both patients with anxious or avoidant attachment patterns could benefit from psychotherapy aimed at identifying and revising insecure working models and transforming them into more secure ones (Daniel, 2006).

## Conclusions

Our findings support the proposition that attachment may be considered as a suitable theoretical framework for understanding variation in QoL and distress experienced by early-stage female breast cancer patients. Sir William Osler (1849–1919), a physician and one of the four founding professors of Johns Hopkins Hospital, who has frequently been described as the father of modern medicine, once said: “it is much more important to know what sort of patient has a disease than what sort of disease a patient has” (John, 2013). Later on, George L. Engel (1977), who acknowledged that each patient has their own story, as well as their own thoughts and feelings, proposed the biopsychosocial model of health and disease and advocated a holistic approach. To this end, attachment theory provides an adequate framework for making patient-centered care front and center.

### Supplementary Information

Below is the link to the electronic supplementary material.

Supplementary file1 (DOCX 31 kb)
