# Correction: The Role of Attachment Styles on Quality of Life and Distress Among Early-Stage Female Breast Cancer Patients: A Systematic Review

**DOI:** 10.1007/s10880-023-09955-3

**Published:** 2023-03-16

**Authors:** Spyridoula Karveli, Petros Galanis, Eirini Marina Mitropoulou, Evangelos Karademas, Christos Markopoulos

**Affiliations:** 1 School of Medicine, National and Kapodistrian University of Athens, 75 Mikras Asias Str, 11527 Athens, Greece; 2 Clinical Epidemiology Laboratory, Faculty of Nursing, National and Kapodistrian University of Athens, Athens, Greece; 3 Department of Psychology, Faculty of Social Sciences, University of Crete, Rethymnon, Greece

**Correction to: Journal of Clinical Psychology in Medical Settings (2023)** 10.1007/s10880-023-09940-w

Due to an oversight by this article’s authors, the orders of authors’ first and last names are given incorrectly in the original publication. The authors’ names should be listed as follows: Spyridoula Karveli, Petros Galanis, Eirini Marina Mitropoulou, Evangelos Karademas, & Christos Markopoulos.

Likewise, the citation should be as follows: Karveli, S., Galanis, P., Mitropoulou, E. M., Karademas, E., & Markopoulos, C. (2023). The role of attachment styles on quality of life and distress among early-stage female breast cancer patients: A systematic review. *Journal of Clinical Psychology in Medical Settings*. https://doi.org/10.1007/s10880-023-09940-w.

