# Supplementary material for: The Role of Attachment Styles on Quality of Life and Distress Among Early-Stage Female Breast Cancer Patients: A Systematic Review
Source: J Clin Psychol Med Settings. 2023 Feb 11;30(4):724–39. doi: 10.1007/s10880-023-09940-w (PMC10560157; doi:10.1007/s10880-023-09940-w)
Supplement: Supplementary file 1 — Supplementary file1 (DOCX 31 kb) [file 10880_2023_9940_MOESM1_ESM.docx]

**Supplementary Material 1**

Search strategy

a. Concept Map for Constructing Search Strategies: Keywords

|  | **Population** | **Iintervention** | **Comparison** | **Outcome** |
| --- | --- | --- | --- | --- |
| **Concepts** | Breast cancer patients | Attachment styles | - | Quality of Life & Distress |
| **MeSH terms** | "Breast Neoplasms/psychology”[Mesh] | "Object Attachment"[Mesh] |  | "Quality of Life”[Mesh], "Stress, Psychological/psychology”[Mesh], "Adaptation,Psychological”[Mesh] “Depression/psychology”[Mesh], "Anxiety/psychology"[Mesh] |
| **Keywords** | breast cancer, breast neoplasm, breast tumor, breast carcinoma, breast cancer survivor* | attachment, adult attachment, attachment style, close relationship, attachment theory, avoidant attachment, anxious attachment, attachment anxiety |  | quality of life, health related quality of life, distress, emotional distress, depression, anxiety, adaptation, post-traumatic growth |

b. Concept Map for Constructing Search Strategies: Keywords

|  | **Concept 1** | **Concept 2** | **Concept 3** | **Concept 4** | **Concept 5** | **Concept 6** | **Concept 7** |
| --- | --- | --- | --- | --- | --- | --- | --- |
|  | “Breast cancer” | Attachment | “Quality of life” | Distress | Depression | Anxiety | Adaptation |
| OR | “Breast neoplasm*” | “Adult attachment” | “Life quality” | “Emotional Distress” | “depressive symptoms” | “anxiety symptoms” | adaptation |
| OR | “Breast tumor*” | “Close relationship” | “Health-Related Quality Of Life” | “Emotional Stress” | “depressive disorder” | “anxiety disorder” | “Psychological Adaptation” |
| OR | “Mammary cancer” | “Avoidant attachment” | HRQOL | Stress |  |  | “Coping Skills” |
| OR | “Malignant Neoplasm of Breast” | “Anxious attachment” |  | “psychological distress” |  |  | “Adaptive Behavior*” |
| OR | “Breast Malignant Tumor” |  |  |  |  |  | “Coping Behavior*” |
| OR | “Breast Carcinoma” |  |  |  |  |  | "emotional adjustment" |

**PubMed**

**Concept #1: breast cancer**

“Breast Neoplasms/psychology”[Mesh] OR “Cancer Survivors/psychology”[Mesh] OR “breast cancer”[tiab] OR “breast neoplasm*”[tiab] OR “breast tumor*”[tiab] OR “breast carcinoma*”[tiab] OR “breast cancer survivor*”[tiab]

**Concept #2: attachment**

“Object Attachment"[Mesh] OR attachment*[tiab] OR "adult attachment*"[tiab] OR “attachment style*"[tiab] OR "close relationship*"[tiab] OR “attachment theory”[tiab] OR "avoidant attachment"[tiab] OR "anxious attachment”[tiab] OR “attachment anxiety”[tiab]

**Concept #3: quality of life**

"Quality of Life"[Mesh] OR “quality of life”[tiab] OR “life quality”[tiab] OR “health-related quality of life”[tiab] OR “health related quality of life”[tiab] OR HRQOL[tiab]

**Concept #4: distress**

"Stress, Psychological/psychology"[Mesh] OR distress[tiab] OR “emotional distress”[tiab] OR “emotional stress”[tiab] OR stress[tiab]

**Concept #5: depression**

"Depression/psychology"[Mesh] OR Depression[tiab] OR “depressive symptom*”[tiab] OR “depressive disorder”[tiab]

**Concept #6: anxiety**

"Anxiety/psychology"[Mesh] OR anxiety[tiab] OR “anxiety symptoms”[tiab] OR “anxiety disorder”[tiab]

**Concept #7: adaptation**

"Adaptation, Psychological”[Mesh] OR adjustment[tiab] OR adaptation[tiab] OR “psychological adaptation”[tiab] OR “coping skills”[tiab] OR “adaptive behavior*”[tiab] OR “coping behavior*”[tiab] OR “coping strategies”[tiab] OR “post-traumatic growth” [tiab] OR social support [tiab] OR “emotional regulation”[tiab]

**Building Block Approach**

#1 AND #2 AND #3

#1 AND #2 AND #4

#1 AND #2 AND #5

#1 AND #2 AND #6

#1 AND #2 AND #7

**SCOPUS**

**Concept #1: breast cancer**

TITLE-ABS-KEY ( "breast cancer"  OR  "breast neoplasm"  OR  "breast tumor"  OR  "breast carcinoma"  OR  "breast cancer survivor*"  OR  "cancer survivor*" )

**Concept #2: attachment**

TITLE-ABS-KEY ( "Object Attachment" OR attachment OR "adult attachment" OR "attachment style" OR "close relationship*" OR "attachment theory" OR "avoidant attachment" OR "anxious attachment" OR "attachment anxiety" )

**Concept #3: quality of life**

TITLE-ABS-KEY ( "quality of life"  OR  "life quality"  OR  "health-related quality of life"  OR  hrqol )

**Concept #4**

TITLE-ABS-KEY ( distress  OR  "psychological distress"  OR  "emotional distress"  OR  "emotional stress"  OR  stress )

**Concept 35**

TITLE-ABS-KEY ( "depression"  OR  "depressive symptom"  OR  "depressive disorder" )

**Concept #6**

TITLE-ABS-KEY ( anxiety  OR  "anxiety symptoms"  OR  "anxiety disorder" )

**Concept #7**

TITLE-ABS-KEY ( adjustment  OR  adaptation  OR  "psychological adaptation"  OR  "coping skills"  OR  "adaptive behavior"  OR  "coping behavior"  OR  "coping strategies"  OR  "post-traumatic growth" )

**Building Block Approach**

#1 AND #2 AND #3

#1 AND #2 AND #4

#1 AND #2 AND #5

#1 AND #2 AND #6

#1 AND #2 AND #7

**EBSCO - PsycInfo**

**Search #1.**

“breast cancer” OR "breast neoplasm*” OR “breast tumor*” OR “breast carcinoma*” OR “breast cancer survivor” OR “cancer survivor*”

AND "object attachment” OR attachment* OR "adult attachment*” OR “attachment style*" OR "close relationship*" OR “attachment theory” OR "avoidant attachment" OR "anxious attachment” OR “attachment anxiety”

AND “quality of life” OR“life quality” OR “health-related quality of life”OR “health related quality of life” OR HRQOL

**Search #2.**

“breast cancer” OR "breast neoplasm*” OR “breast tumor*” OR “breast carcinoma*” OR “breast cancer survivor” OR “cancer survivor*

AND "object attachment” OR attachment* OR "adult attachment*” OR “attachment style*" OR "close relationship*" OR “attachment theory” OR "avoidant attachment" OR "anxious attachment” OR“attachment anxiety”

AND distress OR “emotional distress” OR “emotional stress” OR stress3.

**Search #3.**

“breast cancer” OR "breast neoplasm*” OR “breast tumor*” OR “breast carcinoma*” OR “breast cancer survivor” OR “cancer survivor*

AND"object attachment” OR attachment* OR "adult attachment*” OR “attachment style*" OR "close relationship*" OR “attachment theory” OR "avoidant attachment" OR "anxious attachment” OR “attachment anxiety”

AND depression OR “depressive symptom*” OR “depressive disorder”

**Search #4.**

“breast cancer” OR "breast neoplasm*” OR “breast tumor*” OR “breast carcinoma*” OR “breast cancer survivor” OR “cancer survivor*

AND "object attachment” OR attachment* OR "adult attachment*” OR “attachment style*" OR "close relationship*" OR “attachment theory” OR "avoidant attachment" OR "anxious attachment” OR “attachment anxiety”

AND anxiety OR “anxiety symptoms” OR “anxiety disorder”5.

**Search #5.**

“breast cancer” OR "breast neoplasm*” OR “breast tumor*” OR “breast carcinoma*” OR “breast cancer survivor” OR “cancer survivor*

AND "object attachment” OR attachment* OR "adult attachment*” OR “attachment style*" OR "close relationship*" OR “attachment theory” OR "avoidant attachment" OR "anxious attachment” OR “attachment anxiety”

AND adjustment OR adaptation OR “psychological adaptation” OR “coping skills” OR “adaptive behavior*” OR “coping behavior*”OR “coping strategies” OR “post-traumatic growth” OR “social support” OR “emotion* regulation”

**CINAHL TI & AB**

**Search #1.**

TI ( breast cancer or breast neoplasm or breast carcinoma or breast tumor ) AND TI ( attachment theory or attachment or attachment style ) AND TI ( quality of life or well being or well-being or health-related quality of life )

AB ( breast cancer or breast neoplasm or breast carcinoma or breast tumor ) AND AB ( attachment theory or attachment or attachment style ) AND AB ( quality of life or well being or well-being or health-related quality of life )

**Search #2.**

TI ( breast cancer or breast neoplasm or breast carcinoma or breast tumor ) AND TI ( attachment theory or attachment or attachment style ) AND TI ( adaptation or adjustment )

AB ( breast cancer or breast neoplasm or breast carcinoma or breast tumor ) AND AB ( attachment theory or attachment or attachment style ) AND AB ( adaptation or adjustment )

**Search #3.**

TI ( breast cancer or breast neoplasm or breast carcinoma or breast tumor ) AND TI ( attachment theory or attachment or attachment style ) AND TI ( coping or coping strategies or coping skills or coping behaviour or cope )

AB ( breast cancer or breast neoplasm or breast carcinoma or breast tumor ) AND AB ( attachment theory or attachment or attachment style ) AND AB ( coping or coping strategies or coping skills or coping behaviour or cope )

**Search #4.**

TI ( breast cancer or breast neoplasm or breast carcinoma or breast tumor ) AND TI ( attachment theory or attachment or attachment style ) AND TI stress

AB ( breast cancer or breast neoplasm or breast carcinoma or breast tumor ) AND AB ( attachment theory or attachment or attachment style ) AND AB stress

**Search #5.**

TI ( breast cancer or breast neoplasm or breast carcinoma or breast tumor ) AND TI ( attachment theory or attachment or attachment style ) AND TI social support

AB ( breast cancer or breast neoplasm or breast carcinoma or breast tumor ) AND AB ( attachment theory or attachment or attachment style ) AND AB social support

**Search #6.**

TI ( breast cancer or breast neoplasm or breast carcinoma or breast tumor ) AND TI ( attachment theory or attachment or attachment style ) AND TI (post traumatic growth or post-traumatic growth or post traumatic growth or ptg or posttraumatic growth)

AB ( breast cancer or breast neoplasm or breast carcinoma or breast tumor ) AND AB ( attachment theory or attachment or attachment style ) AND AB ( posttraumatic growth or post-traumatic growth or post traumatic growth or ptg )

**Search #7.**

TI ( breast cancer or breast neoplasm or breast carcinoma or breast tumor ) AND TI ( attachment theory or attachment or attachment style ) AND TI ( distress or anxiety or stress or psychological or depression )

AB ( breast cancer or breast neoplasm or breast carcinoma or breast tumor ) AND AB ( attachment theory or attachment or attachment style ) AND AB ( distress or anxiety or stress or psychological or depression )

**Google Scholar**

| allintitle:"breast cancer" AND "attachment" AND "quality of life" |
| --- |
| allintitle:"breast cancer" AND "attachment" AND "life quality" |
| allintitle:"breast cancer" AND "attachment" AND "health related quality of life" |
| allintitle:"breast cancer" AND "attachment" AND "HRQOL" |
| allintitle:"breast cancer" AND "attachment" AND "stress" |
| allintitle:"breast cancer" AND "attachment" AND "distress" |
| allintitle:"breast cancer" AND attachment AND depression |
| allintitle:"breast cancer" AND attachment AND “depressive symptoms” |
| allintitle:"breast cancer" AND "attachment" AND “anxiety” |
| allintitle:"breast cancer" AND attachment AND “anxiety symptoms” |
| allintitle:"breast cancer" AND attachment AND “anxiety disorder” |
| allintitle:"breast cancer" AND attachment AND adaptation |
| allintitle:"breast cancer" AND attachment AND adjustment |
| allintitle:"breast cancer" AND attachment AND “coping skills” |
| allintitle:"breast cancer" AND attachment AND “adaptive behavior*” |
| allintitle:"breast cancer" AND attachment AND “coping behavior*” |
| allintitle: "breast cancer" AND "attachment" AND "emotional regulation" |
| allintitle: "breast cancer" AND "attachment" AND "social support" |
| allintitle:"breast cancer" AND "attachment security" |
| allintitle:"breast cancer" AND "attachment insecurity" |
| allintitle:"breast cancer" AND "attachment" AND "coping" |
| allintitle:"cancer survivors" AND attachment AND "distress" |
| allintitle:"cancer survivors" AND "attachment" AND "quality of life" |
| allintitle:"cancer survivors" AND "attachment" AND "stress" |
| allintitle:"cancer survivors" AND attachment AND adaptation |
| allintitle:"cancer survivors" AND attachment AND adjustment |
| allintitle: "cancer survivors" AND "attachment" |
| allintitle: "cancer" AND "attachment" AND "quality of life" |
| allintitle: "cancer" AND "attachment" AND "distress" |
| allintitle: "cancer" AND "attachment" AND depression |
| allintitle: "cancer" AND "attachment" AND anxiety |
| allintitle: "cancer" AND "attachment" AND "coping" |
| allintitle: "cancer" AND "attachment" AND "social support" |
| allintitle: "cancer" AND "attachment" AND adjustment |
| allintitle: "cancer" AND "attachment" AND adaptation |
| allintitle: "cancer" AND "attachment" AND "depressive symptoms" |
| allintitle: "cancer" AND "attachment" AND "posttraumatic growth" |

**PMC Europe**

| (TITLE:"breast cancer" AND TITLE:"attachment" AND TITLE:"quality of life") AND (LANG:"eng" OR LANG:"en" OR LANG:"us") |
| --- |
| (TITLE:"breast cancer" AND TITLE:"attachment" AND TITLE:"distress") AND (LANG:"eng" OR LANG:"en" OR LANG:"us") |
| (TITLE:"breast cancer" AND TITLE:"attachment" AND TITLE:"stress") AND (LANG:"eng" OR LANG:"en" OR LANG:"us") |
| (TITLE:"breast cancer" AND TITLE:"attachment" AND TITLE:"depressive symptoms") AND (LANG:"eng" OR LANG:"en" OR LANG:"us") |
| (TITLE:"breast cancer" AND TITLE:"attachment" AND TITLE:"anxiety") AND (LANG:"eng" OR LANG:"en" OR LANG:"us") |
| (TITLE:"breast cancer" AND TITLE:"attachment" AND TITLE:"adaptation") AND (LANG:"eng" OR LANG:"en" OR LANG:"us") |
| (ABSTRACT:"breast cancer" AND ABSTRACT:"attachment" AND ABSTRACT:"quality of life") AND (LANG:"eng" OR LANG:"en" OR LANG:"us") |
| (ABSTRACT:"breast cancer" AND ABSTRACT:"attachment" AND ABSTRACT:"distress") AND (LANG:"eng" OR LANG:"en" OR LANG:"us") |
| (ABSTRACT:"breast cancer" AND ABSTRACT:"attachment" AND ABSTRACT:"depression") AND (LANG:"eng" OR LANG:"en" OR LANG:"us") |
| (ABSTRACT:"breast cancer" AND ABSTRACT:"attachment" AND ABSTRACT:"adaptation") AND (LANG:"eng" OR LANG:"en" OR LANG:"us") |
| (ABSTRACT:"breast cancer" AND ABSTRACT:"attachment" AND ABSTRACT:"adjustment") AND (LANG:"eng" OR LANG:"en" OR LANG:"us") |
| (ABSTRACT:"breast cancer" AND ABSTRACT:"attachment" AND ABSTRACT:"posttraumatic growth") AND (LANG:"eng" OR LANG:"en" OR LANG:"us") |
| (ABSTRACT:"breast cancer" AND ABSTRACT:"attachment" AND ABSTRACT:"coping strategies") AND (LANG:"eng" OR LANG:"en" OR LANG:"us") |
| (ABSTRACT:"cancer survivors" AND ABSTRACT:"attachment" AND ABSTRACT:"quality of life") AND (LANG:"eng" OR LANG:"en" OR LANG:"us") |
| (ABSTRACT:"cancer survivors" AND ABSTRACT:"attachment" AND ABSTRACT:"distress") AND (LANG:"eng" OR LANG:"en" OR LANG:"us") |
| (ABSTRACT:"cancer survivors" AND ABSTRACT:"attachment" AND ABSTRACT:"depression") AND (LANG:"eng" OR LANG:"en" OR LANG:"us") |
| (ABSTRACT:"cancer survivors" AND ABSTRACT:"attachment" AND ABSTRACT:"anxiety") AND (LANG:"eng" OR LANG:"en" OR LANG:"us") |
| (ABSTRACT:"cancer survivors" AND ABSTRACT:"attachment" AND ABSTRACT:"adjustment") AND (LANG:"eng" OR LANG:"en" OR LANG:"us") |
| (ABSTRACT:"cancer survivors" AND ABSTRACT:"attachment" AND ABSTRACT:"social support") AND (LANG:"eng" OR LANG:"en" OR LANG:"us") |
| (ABSTRACT:"cancer survivors" AND ABSTRACT:"attachment" AND ABSTRACT:"posttraumatic growth") AND (LANG:"eng" OR LANG:"en" OR LANG:"us") |
| (ABSTRACT:"cancer survivors" AND ABSTRACT:"attachment security") AND (LANG:"eng" OR LANG:"en" OR LANG:"us") |
| (ABSTRACT:"cancer survivors" AND ABSTRACT:"insecure attachment") AND (LANG:"eng" OR LANG:"en" OR LANG:"us") |
| (ABSTRACT:"cancer survivors" AND ABSTRACT:"attachment") AND (LANG:"eng" OR LANG:"en" OR LANG:"us") |
